# TransOral endoscopic UltraSonic Surgery (TOUSS): a preliminary report of a novel robotless alternative to TORS

**DOI:** 10.1007/s00405-014-3423-6

**Published:** 2014-12-16

**Authors:** Mario M. Fernández-Fernández, Lourdes Montes-Jovellar, Pablo Luis Parente Arias, Primitivo Ortega del Alamo

**Affiliations:** 1 Department of Otolaryngology, Hospital Universitario del Henares, Avda Marie Curie s/n, 28822 Coslada, Madrid Spain; 2 Otolaryngology Head & Neck Surgery Department, MD Anderson International, c/Arturo Soria 270, 28033 Madrid, Spain; 3 Department of Otolaryngology, Hospital Universitario de A Coruña, As Xubias s/n, 15006 A Coruña, Spain; 4 Department of Otolaryngology, Hospital Universitario de Móstoles, c/Río Júcar s/n, 28935 Mostoles, Madrid Spain

**Keywords:** Transoral surgery, Oropharyngeal carcinoma, Hypopharyngeal carcinoma, Supraglottic carcinoma, Robotic surgery, Thunderbeat

## Abstract

The objective of this study is to describe and evaluate the feasibility of TransOral UltraSonic Surgery (TOUSS), a new endoscopic alternative to transoral robotic surgery for approaching pharyngeal and laryngeal tumours based on ultrasonic scalpel as a resection tool. This is a prospective study on 11 consecutive patients with pharyngeal and supraglottic carcinomas between December 2013 and August 2014. All tumours were resected transorally with 35 cm ThunderbeatTM. Exposure was achieved using GyrusTM FK-retractor and Olympus ENDOEYE Flex 5 mm 2D/10 mm 3D deflecting tip video laparoscopes. We evaluated tumour staging, surgical margins, surgical time, blood transfusions, tracheostomy, enteral feeding, postoperative pain and hospital stay. The operating room setup and procedure are described. This series comprised seven early and four locally advanced carcinomas. The mean setup for TOUSS and resection time were 16 and 70.9 minutes. No major intraoperative complications were identified. The average time of nasogastric feeding tube dependence (*n *= 9) was 13 days. Gastrostomy was performed in one patient. The average hospital stay was 14.3 days. Postoperative pain was satisfactory treated with nonsteroidal anti-inflammatory drugs. We have described TOUSS as a new feasible and intuitive procedure to approach endoscopically pharyngeal and supraglottic tumours, with good intraoperative conditions and functional outcomes.

## Introduction

Transoral robotic surgery (TORS) has demonstrated its feasibility, high rates of local control and good functional outcomes for lesions of oral cavity, oropharynx and laryngopharynx [1–4]. High definition videocameras as well as the new videoendoscopes have a critical role in its results. In fact, TORS represents a step forward in the endoscopic way to treat pharyngeal and laryngeal lesions. However, more affordable proposals are needed regarding the high costs of robotic surgery, in order to spread the endoscopic transoral approach philosophy.

Many papers have been published about the safety, utility and advantages of the ultrasonic scalpel [5]. It has been used routinely in surgical settings such as laparoscopic surgery and open abdominal and thoracic procedures in the last two decades. Specifically in head and neck surgery, it has been widely used in the last decade for open and minimally invasive thyroidectomy, and showing its potential for other open head and neck procedures like glossectomy, tonsillectomy or laryngopharyngectomy [6–8]. Its superior haemostasis allows clean and bloodless procedures, and the lower temperature and heat diffusion to surrounding tissue improve the safety compared with electrocautery [5, 9].

This paper describes a novel endoscopic approach, TransOral Ultrasonic Surgery (TOUSS), to treat laryngopharyngeal lesions, combining ultrasonic energy for cutting and coagulating, and high definition 2D–3D endoscopic imaging, in order to reach the same output of TORS.

## Materials and methods

A protocol to treat human subjects with ultrasonic scalpel through endoscopic approach was designed and approved by our institutional review board. The inclusion criteria were: (1) at least 18 years old, (2) pharyngeal or laryngopharyngeal or supraglottic neoplasm with the indication for surgical excision (3) consent for transoral surgical treatment with ultrasonic scalpel. Exclusion criteria were (1) pregnancy, (2) unable to understand the surgical procedure (3) previous treatment of the laryngopharyngeal neoplasm. All patients were counselled about the alternatives to TOUSS and all of them consented to endoscopic surgical treatment of their laryngopharyngeal cancer.

### Laryngopharyngeal retractor

The adequate exposition of the pharynx and the larynx was achieve through Gyrus^®^ FK-retractor (Gyrus Medical Inc., Maple Grove, Minnesotta) as it is used for TORS.

### Endoscopic vision

**Fig. 1:**
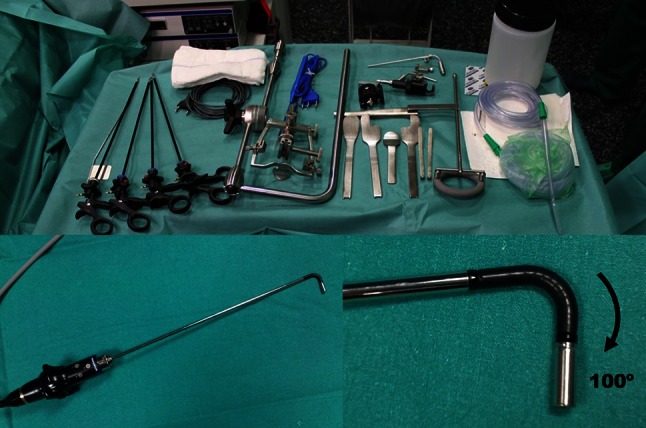
Surgical instruments including the Gyrus FK-retractor, the scope holder, a set of laparoscopic forceps and scissors. At the *bottom*, the videolaparoscope and the deflectable tip bended at maximum are shown

The endoscopic vision was achieved through both Olympus ENDOEYE Flex 5 mm 2D or ENDOEYE Flex 10 mm 3D videolaparoscopes (Olympus Medical System Corp, Tokyo, Japan). The deflectable tip allows a refinement of the surgical vision with small movements of the joysticks at the camera head, up to 100° field of view in all directions. The videoendoscope and the set of laparoscopic instruments are shown in Fig. 1.

### Ultrasonic scalpel

The ultrasonic scalpel Thunderbeat^®^ (Olympus Medical System Corp, Tokyo, Japan) was used as cutting and coagulating instrument. The ultrasonic energy allows cutting and coagulating tissues simultaneously with relatively low heat and lateral thermal injury. The basic effect is similar to electrosurgery or lasers, denaturing proteins, but the mechanism of ultrasonic energy consists in transferring to the tissue the vibrating mechanical energy at high frequency (25–55 kHz), breaking hydrogen bonds at a low range of temperature compared with electrocautery or laser (200 °C maximum temperature vs 400 °C). The cutting mechanism is achieved by a sharp blade over a distance of 100 μm. The precision of cutting and coagulation can be controlled by the surgeon by adjusting the power level, and lateral thermal damage is limited due to the lower working temperature. Additionally, Thunderbeat^®^ incorporates a bipolar vessel sealing system that can be activated separately; so the possibility of additional sealing lines improves the confidence with vessels up to 7 mm like the lingual or upper laryngeal artery that are frequently exposed [5]. The Thunderbeat^®^ 5 mm 35 cm shaft length allows a comfortable resection in terms of working distance.

**Fig. 2:**
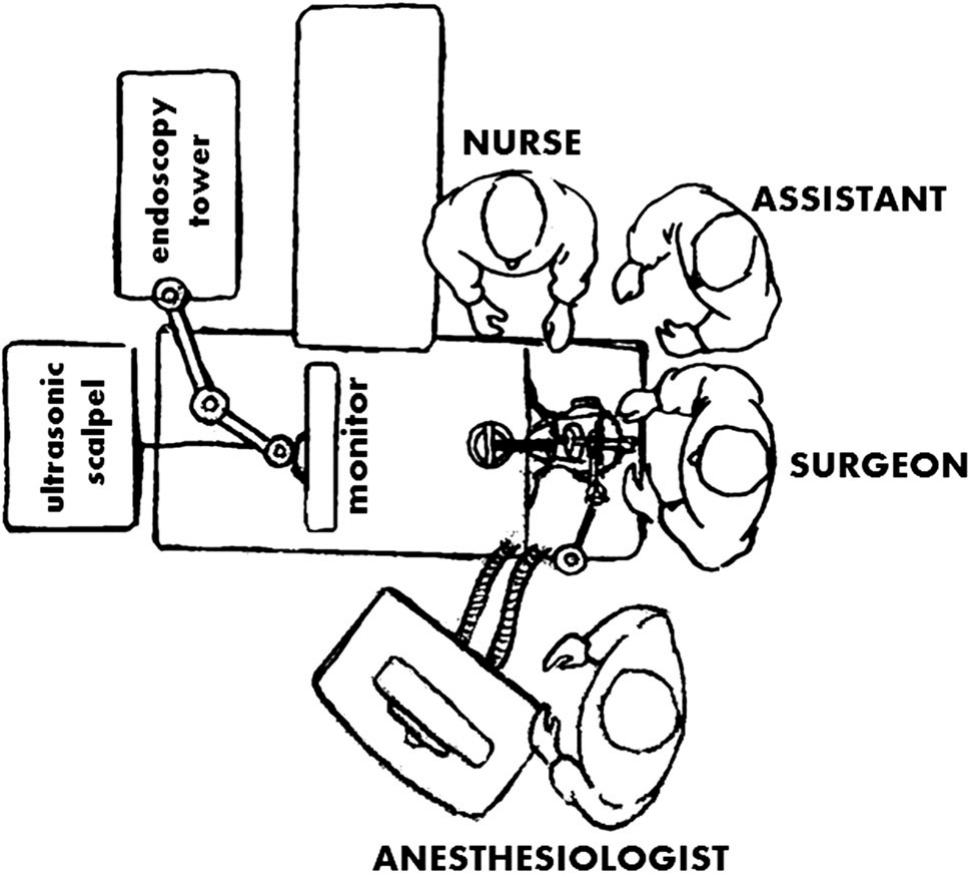
Illustration showing the OR setup for TOUSS

**Fig. 3:**
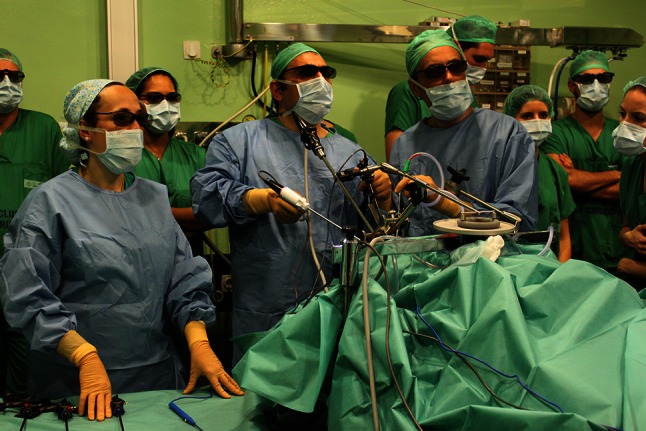
TOUSS setup: the surgeon and assistant are standing up at the head of the patient. The scope holder arm is attached to the left side of the surgical bed. All the surgical team need to wear 3D glasses to watch the procedure with 3D endoscopy

**Fig. 4:**
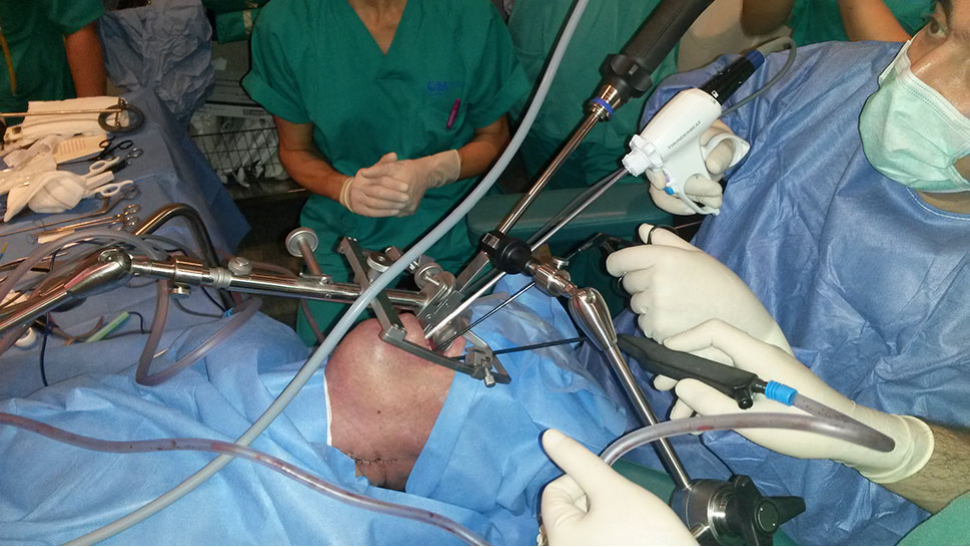
Showing a closer view of TOUSS setup, the use of ultrasonic scalpel for tumoral resection and the assistant by the left side of the surgeon keeping clear the endoscopic vision with a suction cannula

After general anaesthesia, with the patient in supine position, the articulated arm scope holder is attached to the left side of the surgical bed and the chest support platform to the right side. The videolaparoscope is fitted into a scope holder. The monitor is place at the feet of the OR table, as well as the Thunderbeat generator (Figs. 2, 3). Tumoral resection is done under endoscopic vision, keeping the mobile jaw against the mucosa in order to reduce its damage due to direct contact with the vibrating shaft (Fig. 3). A long suction cannula is hold by the assistant to avoid the smoke overclouding the endoscopic vision when the ultrasonic device is activated (Fig. 4).

Neck dissection was performed simultaneously and prior to tumoral resection. This aspect is critical when a cervicopharyngeal communication is expected, in order to protect the carotid artery and prevent its accidental damage. Neck closure was delayed until the end of the procedure to facilitate the pharyngeal closure from outside if it was necessary.

In this study we collected data about tumour site, histology, clinical and pathological stage using AJCC criteria, TOUSS primary tumour removal operative time, TOUSS setting time, tumoral margins, tracheostomy, nasogastric or gastrostomy feeding tube, blood transfusions, hospitalization time, perioperative complications, and days of feeding tube dependence. In our institutions, carcinoma at the margin is considered a positive margin; less than 5 mm, a close margin; and 5 mm or more, a clear margin. The patients were followed to assess the control of postoperative pain (0—no medication needed, 1—mild: pain controlled with 1 nonsteroidal anti-inflammatory drug, 2—moderate: pain controlled with combination of nonsteroidal anti-inflammatory drugs, and/or addition of steroids, 3—severe: pain controlled with opiates; 4—uncontrollable pain). The data analysis was done with Microsoft Excel (Microsoft Corp, Redmond, WA).

## Results

A prospective study of 11 consecutive patients who underwent TOUSS was done. All procedures were performed by the first author in four institutions. The most important data are summarized in Table 1.

**Table 1:** Showing most important data

Case	Institution	Tumour site	TOUSS setup (min)	Endo vision	Resect time (min)	Trach (days)	NG depend (days)	PTNM	Hospital stay (days)	Pain control	Cervico pharinx Comm.	Postop complic
1	HUM	Hypopharynx Lateral wall Pyriform sinus	32	2D	150	10	10	T3N0M0 Est. III	10	1	No	No
2	HUM	Larynx Supraglottic Valleculae	25	2D	120	28	28	T3N0M0 Est. III	30	2	No	Postop bleeding (CO_2_ laser resection area)
3	HUH	Oropharynx Posterior-lateral Hypopharyngeal ext	12	2D	120	+ (still open)	Gastrostomy	T2N1M0 Est. III	43	2	Yes	No
4	HUH	Supraglottic Supraglottis Pyriform sinus	17	2D	104	−	14	T2N0M0 Est. II	14	2	No	No
5	HUM	Hypopharynx Pyriform sinus	13	2D	64	−	13	T2N0M0 Est. II	13	2	No	No
6	MDA	Oropharynx Soft palate Posterolateral	15	3D	92	TL	21	T4bN0M1 Est. IV 2nd primary	14	2	Yes	Left internal jugular vein thrombosis
7	HUH	Oropharynx Soft palate Tonsil	15	3D	32	TL	−	T1N0M0 Est. I	1	1	No	No
8	HUC	Oropharynx Tonsil	25	3D	18	−	10	T2N2bM0 Est. IV	10	2	Yes	Postop bleeding
9	MDA	Oropharynx Tongue base	8	2D	27	−	3	T1N2cM0 Est. IV	10	2	No	No
10	HUH	Oropharynx Tonsil Tongue base	10	2D	45	7	5	T1N0M0 Est. I 3rd primary	9	1	Yes	No
11	MDA	Oropharynx/ Oral cavity Post oral tongue Tongue base	5	2D	8	0	−	T1N0M0 Est. I	3	2	No	No

Pain control 0—no medication needed, 1—mild: pain controlled with 1 nonsteroidal anti-inflammatory drug, 2—moderate: pain controlled with combination of nonsteroidal anti-inflammatory drugs, and/or addition of steroids, 3—severe: pain controlled with opiates, 4–uncontrollable pain

*HUM* Hospital Universitario Mostoles, *HUH* Hospital Universitario Henares, *MDA* MD Anderson Internacional, *HUC* Complejo Hospitalario Universitario A Coruña, *Min* minutes, *Endo* *vision* endoscopic vision, *Resect time* resection time, *Trach.* tracheostomy, *NG depend* nasogastric tube dependence, *Blood trans* blood transfusion, *Cervico pharinx comm* cervicopharyngeal communication, *Postop complic* postoperative complications, *TL* previous total laryngectomy

Ten male and one female patients with a mean age of 60.6 years (range 46–69) were treated between December 2013 and August 2014. All patients had a history of smoking for at least 30 years (average 40.1; range 30–50) and drinking. Tumour sites were supraglottic (*n* = 2; 18.2 %), oropharynx (*n* = 6; 54.5 %), hypopharynx (*n* = 2; 18.2 %), oro-hypopharynx (*n* = 1; 9.1 %). Six patients had an advanced stage III–IV carcinoma (54.6 %) and five stage I–II (45.4 %); four (36.4 %) T3–T4 and seven (63.7 %) T1–T2 carcinomas. 90.9 % were treated endoscopically with TOUSS exclusively. One patient (case #2) was treated combining an endoscopic approach (TOUSS) and a microlaryngoscopic approach using CO_2_ laser due to the proximity of the vocal cords to the inferior aspect of the lesion. Most patients were treated with curative intention; patient #6 had a pulmonary metastasis (with good response to cetuximab) and the indication for surgery was set in multidisciplinary meeting to control symptoms of a locally advanced second primary tumour. Three cases (27.3 %) were second primary tumours, all of them had already undergone bilateral functional neck dissection during previous surgery, and adjuvant radiotherapy; unilateral or bilateral neck dissection was performed on the other eight patients (72.7 %). No major intraoperative complications were identified. Pharyngocervical communication was not considered a complication since it was mandatory in order to achieve a safe surgical margin. Five patients were considered at risk of pharyngocervical communication, due to the deep extension of the tumour and it occurred in four. All were successfully managed with direct transoral suture. An sternocleidomastoid muscle flap was used in cases #3 and 10, to reinforce the pharyngeal suture line; one free radial forearm flap was elevated to reconstruct soft palate and lateral pharyngeal wall in case #6 that failed in day 4 due to thrombosis of the internal jugular vein secondary to a postoperative worsening of the patient neck lymphedema (secondary to previous radiotherapy). In this patient, the cervicopharyngeal communication was successfully closed with direct suture and soft palate reconstruction was delayed. No blood transfusions were necessary for any patient at no time. TOUSS mean setup was 16 min (range 5–32). The average resection time was 70.9 min (range 8–150). The tumour was fragmented in three patients (27.3 %; cases #1, #2 and #6), in order to allow an adequate visualization of the inferior resection. The other eight tumours (72.7 %) were resected en bloc. The surgical margin was negative for ten patients (90.9 %), and it was uncertain for the patient #6 treated with palliative intention. One patient (case #3) had perineural invasion, so adjuvant radiotherapy of the primary tumour was indicated. Neck dissection was performed always prior to primary tumoral resection. 8 patients (72.7 %) underwent a neck dissection, and positive nodes were founded in three of them (37.5 %; *n* = 8). Postoperative complications were registered in three patients (27.3 %): bleeding coming from the anterior commissure (case #2) in day 1 after surgery (this area was resected with CO_2_ laser), an oral bleeding coming from the tonsillar area (case #8) in day 5, both of them successfully controlled in the OR; and the internal jugular vein thrombosis referred on patient #6. Two patients had already a total laryngectomy. Four preventive tracheostomies (44.5 %; *n* = 9) were performed in the other nine patients, due to the extension of the local resection (case #3), bad pulmonary conditions (cases #1 and 2), and difficult intubation (case #10). Three of them were closed within 1 month (days 7, 10 and 28), and one patient (case #4) is keeping the tracheostomy opened until the end of the adjuvant radiotherapy of the primary site. So tracheostomy was avoided in five patients. Excluding total laryngectomy patients, 100 % of locally advanced tumours needed a preventive tracheostomy, but only 16.5 % of the early primary lesions. Nasogastric feeding tube was inserted in nine patients (81.8 %) for an average of 13 days (range 3–28 days). When a cervicopharyngeal communication was observed, oral feeding was delayed in most patients until day 10–14. No complications related to aspiration were registered. The nasogastric feeding tube was replaced with a gastrostomy in patient #6 before starting radiotherapy of the primary site. Patients #7 and 11 didn’t need nasogastric feeding tube. The average hospital stay was 14.3 days (range 1–43). Postoperative pain was considered mild or moderate for all patients as it was successfully treated with one intravenous nonsteroidal anti-inflammatory drug in three patients (27.3 %) or a combination of two in eight (72.7 %). The need of opiate medication was not observed in any patient.

## Discussion

There are an increasing number of papers reporting better functional outcomes of TORS compared with both open surgical techniques and chemoradiotherapy [10]. It is clear that transoral endoscopic approach can be a step forward and the endoscope represents a real alternative to microscope for minimally invasive approach of upper aerodigestive tract lesions. But TORS is unreachable for most of ENT departments and there is not even evidence of its cost-effectiveness [11, 12]. We have designed TOUSS as a “robotless” endoscopic transoral procedure, inspired in laparoscopic setup, in order to get, at least, the same output as reported for TORS. It is mandatory to compare microscopic laser surgery with any transoral technique for laryngopharyngeal tumours. However, the endoscope means a different philosophy as it offers the possibility to enter “into the room” instead of keeping “outside the room”, avoiding the need of an adequate exposition from outside the patient, as it is required for laser surgery. So our first step was to compare our results with TORS as an endoscopic procedure. Larger series of patients will demonstrate if endoscopic approach is superior to microscopic transoral approach. Shiotani has reported an experience with the same endoscopic philosophy, mainly for T1–T2 supraglottic and hypopharyngeal carcinomas, using electrocautery instruments [13]. Our design is based on ultrasonic energy as the resection tool, as the way to get a clean and safe transoral endoscopic resections of any pharyngeal and laryngeal lesion with an optimal control of the surgical margin.

Ultrasonic energy has been already used for open surgical applications in oral cavity, oropharynx, larynx and pharynx [6–8] with good oncological and functional results. Additionally TOUSS allows direct manipulation of the tissue, so the surgeon can keep the tactile input. The advantage of deflectable tip endoscopes is the easy refinement of the endoscopic visualization field with the endoscope joystick. However we cannot get conclusions about the indications for 3D endoscopic vision, but it seems that it can offer superior spatial orientation for those cases at risk of cervicopharyngeal communication.

As well as for TORS [14], indications for TOUSS could be extended to locally advanced pharyngeal tumours. In fact, four patients (36.4 %) were candidates for a mandibulotomy if an open technique were planned. The OR setup for TOUSS was as quick as it has been published for TORS [4, 11]. We have observed a setting up time as low as 5 min after 11 cases, which is lower than the 10 min average time reported by Aubry et al. [12] for the robot setup after their first ten cases of TORS. An average of 70.9 min is already a reasonable resection time, and even a little lower than results reported by Park after 39 oropharyngeal carcinomas [15]. The reposition of the endoscope during TOUSS in order to refine the endoscopic view was identified as the most time consuming aspect of the procedure. The experience with the deflectable endoscope setup is a critical point of the learning curve.

Thunderbeat^®^ jaws are still too bulky for working close to vocal cords when supraglottic lesions are too close to them. So until we introduce a more fine instrument, we are keeping using microscopic laser surgery only for such situation (case #2), as it was described for TORS by other authors [16].

A fast swallowing recover has been reported for TORS [15, 17], with better swallowing results compared with chemoradiation [3, 4]. Only patient #4, with wide pharyngeal resection, keeps the tracheostomy and a gastrostomy. Return of oral feeding was possible in the other ten patients. Park had reported an average of 8.1 days of nasogastric feeding tube dependence (range 2–14) for hypopharyngeal squamous cell carcinomas [18] and 6 days for oropharyngeal carcinomas treated with TORS [14]. Genden has published an average time before starting oral intake as low as 1–3 days [19]. So this aspect is very dependent on each particular institution protocol and experience. In fact, Boudreaux et al. [20] have reported a hospital stay of 17 days. In our series, oral intake was started before discharge from hospital in all patients, and delayed to day 10–14 when a cervicopharyngeal communication had to be repaired; only patient #10 with a small communication was considered for an earlier oral intake. The average nasogastric feeding tube dependence and hospital stay in our series was 13 and 14.3 days respectively. We avoid discharging patients until the nasogastric feeding tube could be removed and tracheostomy could be safely closed. Only one postoperative bleeding complication was attributable directly to the ultrasonic scalpel, in patient #6, but successfully controlled in the OR. Postoperative pain was satisfactory relieved with one intravenous nonsteroidal anti-inflammatory drug or a combination of two.

In conclusion, we have described TOUSS as a new feasible, intuitive and affordable procedure to approach endoscopically pharyngeal and laryngeal tumours, even for locally advanced carcinomas, with good functional outcomes. TOUSS is a promising way to easily spread the philosophy of the endoscopic approach to the pharynx and the larynx.
